# Neural Correlates of Long-Term Memory Enhancement Following Retrieval Practice

**DOI:** 10.3389/fnhum.2021.584560

**Published:** 2021-02-04

**Authors:** Eugenia Marin-Garcia, Aaron T. Mattfeld, John D. E. Gabrieli

**Affiliations:** ^1^Department of Brain and Cognitive Sciences and McGovern Institute for Brain Research, Massachusetts Institute of Technology, Cambridge, MA, United States; ^2^Faculty of Psychology, University of the Basque Country (UPV/EHU), Donostia-San Sebastian, Spain; ^3^Ikerbasque, Basque Foundation for Science, Bilbao, Spain; ^4^Department of Psychology, Florida International University, Miami, FL, United States

**Keywords:** cue recall, fMRI, long-term memory, retrieval practice, testing effect

## Abstract

Retrieval practice, relative to further study, leads to long-term memory enhancement known as the “testing effect.” The neurobiological correlates of the testing effect at retrieval, when the learning benefits of testing are expressed, have not been fully characterized. Participants learned Swahili-English word-pairs and were assigned randomly to either the Study-Group or the Test-Group. After a week delay, all participants completed a cued-recall test while undergoing functional magnetic resonance imaging (fMRI). The Test-Group had superior memory for the word-pairs compared to the Study-Group. While both groups exhibited largely overlapping activations for remembered word-pairs, following an interaction analysis the Test-Group exhibited differential performance-related effects in the left putamen and left inferior parietal cortex near the supramarginal gyrus. The same analysis showed the Study-Group exhibited greater activations in the dorsal MPFC/pre-SMA and bilateral frontal operculum for remembered vs. forgotten word-pairs, whereas the Test-Group showed the opposite pattern of activation in the same regions. Thus, retrieval practice during training establishes a unique striatal-supramarginal network at retrieval that promotes enhanced memory performance. In contrast, study alone yields poorer memory but greater activations in frontal regions.

## Introduction

Testing measures what we know, but can also be an effective learning method itself. The benefit of testing, or retrieval practice, over repeated study has been called the “testing effect” (Abbott, 1909; Karpicke and Roediger, 2008). The testing effect is well established, generalizes across ages, materials, and test formats (Carpenter and DeLosh, 2006; Roediger and Smith, 2012; Dulonsky et al., 2013; Pan and Rickard, 2018), and is more beneficial than elaborative encoding (Karpicke and Blunt, 2011). Two main hypotheses have been investigated related to the testing effect. According to one, the testing effect is associated with strengthening links between cues and targets creating more effective mediators during retrieval (Pyc and Rawson, 2010). Accordingly, failure to retrieve during the test may induce a search process for better mediators during subsequent study opportunities. Alternatively, but not mutually exclusive, it has been proposed that the testing effect results from the enhancement of retrieval-related processes, for example, memory search (Karpicke and Blunt, 2011). Supporting this claim, the testing effect is more robust when test-trials involve more effortful retrieval (e.g., recall vs. recognition), even when the final test is in a different format (Carpenter and DeLosh, 2006). The brain mechanisms of the testing effect have been examined during *encoding* or the learning of material (Nelson et al., 2013), but the brain basis of the testing effect during the *retrieval* of information from long-term memory is less well understood. Indeed, it is the superior long-term memory retrieval of information that is the critical expression of the “testing effect.” The goal of the present study was to discover the neural correlates of the retrieval of long-term memories that have been enhanced by the testing effect.

Studies examining the encoding phase of test-effect paradigms have demonstrated that the long-term retention advantage attributed to retrieval practice is based on the enhancement of cognitive processes that involve both memory successes at encoding (i.e., strengthening associations between cues and responses) and at retrieval (i.e., memory search processes). Activations in the anterior cingulate cortex (Eriksson et al., 2011), middle temporal gyri (Van den Broek et al., 2013), anterior hippocampus, lateral temporal neocortex, and medial prefrontal cortex (Wing et al., 2013) have all been related to encoding differences in individuals who practiced both study and retrieval vs. repeated study only. Parietal cortex activations during the encoding of test-effect paradigms have consistently been correlated with successful long-term retention (Eriksson et al., 2011; Nelson et al., 2013; Van den Broek et al., 2013; Wing et al., 2013; Keresztes et al., 2014; Liu et al., 2014; Vestergren and Nyberg, 2014; Wirebring et al., 2015). One study has provided a potential mechanistic understanding of parietal cortex contribution to repeated testing whereby greater variability in patterns of activation in parietal cortex across retrieval tests was associated with subsequent memory success (Wirebring et al., 2015). Activations in the basal ganglia have also been observed during encoding (Van den Broek et al., 2013).

The neural correlates of the “testing effect” at retrieval, when the benefits of the “testing effect” are apparent, have been examined much less. Two studies observed greater activations related to memory enhanced by the testing effect in parietal, frontal, insular, temporal, and thalamic regions (Keresztes et al., 2014; Wirebring et al., 2015). However, due to differences in experimental design and approach, limited conclusions from these studies can be drawn. For example, Wirebring et al. (2015) evaluated whole-brain activations for repeatedly tested word pairs that were subsequently remembered vs. forgotten but did not compare tested vs. studied-only word pairs that were correctly recalled. The other study compared repeatedly tested vs. studied-only word pairs but constrained their analyses to regions of interest defined by a working memory task (Keresztes et al., 2014). Thus, the neural differences for the testing effects during retrieval, which is defined by the difference between learning that combines study and testing in comparison with learning that only includes repeated study, remains poorly understood.

We aimed to discover the distinct neural correlates of long-term memory retrieval after repeated study vs. a mixture of study and retrieval practice, with overall exposure held constant. We predicted that the testing effect enhances memory by strengthening relevant associations by engaging additional neurobiological systems and altering cognitive control efforts during retrieval (Van den Broek et al., 2016). Young adult participants were assigned randomly to either a Study condition comprised of only the repeated study of Swahili–English word-pairs or a Test condition comprised of an equal number of training exposures but with half exposures involving a practice cued-recall test. After a 1-week retention period, all participants had a final cued-recall test in the scanner. We examined if successful learning, after a mixture of studying and testing, was associated with better memory performance and similar or dissimilar retrieval-related brain activations compared to individuals who only studied the same materials. A question of interest was whether testing-related superior memory reflects differential activation within brain regions typically activated for memory retrieval that supports the strengthening of the memory representation or whether the superior memory associated with retrieval practice involves the inclusion of these regions with additional brain regions associated with other memory systems.

## Materials and Methods

### Participants

Volunteers (*n* = 43) were recruited from the local Cambridge community. Two participants (one from each group) were excluded from further analysis for excessive motion resulting in a total of 41 participants (26 females), between the ages of 18 and 31 years (age mean ± SD, 21.9 ± 3.9). Participants were randomly assigned to either a Study group (*n* = 20, 12 females) or a Test group (*n* = 21, 14 females). We used a between-subject design based on previous behavioral pilots to maximize the behavioral testing effect. All participants were right-handed, native English speakers, with no knowledge of Swahili, with normal or corrected-to-normal vision, and without any history of developmental, neurological, or psychiatric disorders. Participants were compensated for their time at the rate of $20/h for behavioral sessions and $30/h for scanning sessions. Before beginning the experiment, informed consent was obtained from all participants, as required by the Massachusetts Institute of Technology Committee on the Use of Humans as Experimental Subjects.

### Materials

Sixty Swahili–English vocabulary pairs (e.g., theluji—snow; Nelson and Dunlosky, 1994) were used in this experiment, with the known English word being the translation of the unknown Swahili word. All the words were nouns with at least three letters. Stimuli were presented in the center of the screen in white, 40-point Arial font on a gray background. The presentation was coded with PsychoPy2 Experiment Builder (Peirce, 2007).

### Experimental Procedure

The experiment took place over 2 days separated by 1 week. On the first day, participants in the Study Group viewed 60 word-pairs across eight consecutive study runs, while participants in the Test Group had four study and four test runs in alternating order. Thus, participants in both groups had equivalent exposure to the word-pairs. Word-pairs were randomly organized in groups of four. Each group was preceded by either the cue “STUDY” during study runs or “TEST” during test runs. Cues were presented for 2 s. Word-pairs during study and test runs were presented for 4 s. During study runs, participants were instructed to read the Swahili–English word-pairs aloud. During test runs, participants were shown the Swahili word and the first letter of the English word and were required to read the Swahili word and perform a cued recall test for the English word. Word-pairs order was randomized across each run. If they could not remember the English word, they were asked to say, “forget.” No explicit feedback was given during test runs, thus the full word pair was not present during the test runs but participants had the chance to check their previous performance in subsequent study periods. The main instruction was to try to learn all word-pairs for the final test 1 week later but participants were not informed of the final test format during training.

After a week delay, participants returned and performed a cued recall test for all the word-pairs, randomly ordered, and with no feedback. During this session, we measured the blood oxygen level-dependent (BOLD) functional magnetic resonance imaging (fMRI) response when participants were performing the test. The scanning session was broken into three runs of 20 word-pairs each. Each run lasted 6 min and 40 s. We used an Apple Macintosh laptop running Psychopy software (Peirce, 2007) to display the stimuli and control experimental timing. Stimuli were projected through a wave-guide on a rear projection screen (Da-Lite) and participants viewed the screen using a mirror attached to the head coil.

**Figure 1:**
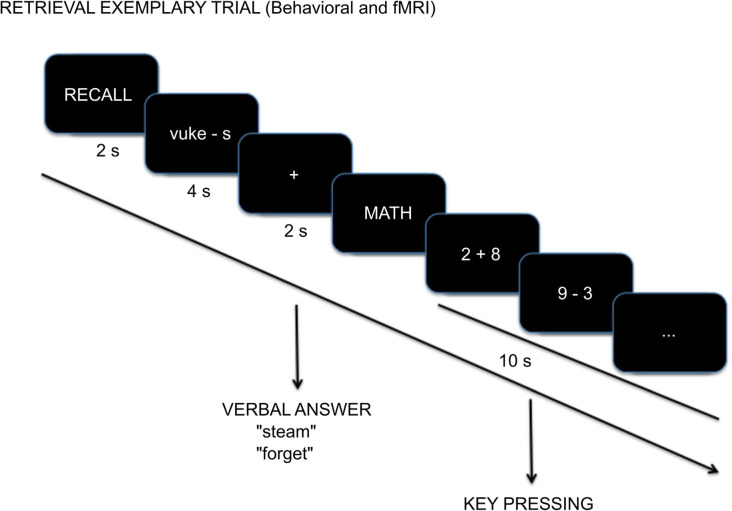
Example cued-recall trial with timing details during the Test session.

A trial began with the “RECALL” instruction presentation (2 s). Then the Swahili word and the first letter of the English translation (e.g., “vuke-s”) were presented for 4 s during which participants tried to remember the English word without responding. A fixation-cross (2 s) followed and as soon as it appeared, participants were instructed to say the English word (e.g., “steam”) or if they could not remember the translation, to say “forget.” Reaction time data is not reported because there was a preparatory period of 4 s related to fMRI acquisition requirements that does not facilitate a true measure of reaction time. The verbal answer was recorded with Audacity (The Audacity Team, 2012). Trials ended with a non-mnemonic distractor task that began with the “MATH” instruction screen (2 s) followed by five arithmetic exercises, sums or subtractions, 2 s each. Participants evaluated whether the result was bigger than 5 by pressing buttons on an MR compatible button box with the right hand (index finger for “yes” and middle finger for “no”; Figure 1). This task was used as an active baseline with minimal recollection processes (Stark and Squire, 2001) so that there was a baseline that was unlikely to include any sort of retrieval practice.

### fMRI Data Acquisition and Analysis

Imaging data were acquired on a 3.0T Siemens Magnetom Tim Trio scanner using a 32-channel phased-array head coil. To help stabilize head position, participants were provided with a foam pillow. Participants used earplugs to reduce scanner noise. A scanner safe microphone was installed in the scanner bed to record the responses. A whole-head, magnetization-prepared rapid gradient echo (MPRAGE), T1-weighted, anatomical image was obtained prior to the functional runs (acquisition parameters: TR = 2,530 ms, TE = 1.61 ms, flip angle = 7°, voxel resolution = 1 × 1 × 1 mm, FOV = 256 × 256 mm, 176 sagittal slices).

Three functional runs were collected using T2*-weighted gradient-echo echo-planar imaging (EPI) scans (acquisition parameters: TR = 2,200 ms, TE = 30 ms, flip angle = 90°, voxel resolution = 3.125 × 3.125 × 3.3 mm, FOV = 64 × 64 mm, 36 axial slices providing whole brain coverage for 182 acquisitions). The first four volumes of each functional run were discarded to allow for stabilization of longitudinal magnetization.

#### MRI Preprocessing

Standard preprocessing was implemented in Nipype v0.7 (Gorgolewski et al., 2011) using tools from FSL v5.0 (Smith et al., 2004), Analysis of Functional Neuroimages (AFNI; Cox, 1996), FreeSurfer 5.1.0 (Dale et al., 1999), and artifact detection (ART). Data were converted from Siemens Dicom format to nifti file format using the *mri_convert* command from FreeSurfer. Surface reconstruction and subcortical segmentation were performed by FreeSurfer and verified *via* visual inspection. Simultaneous slice timing and motion correction were run through the Nipy algorithm using default parameters realigning all functional volumes to the first volume of the first functional run using a rigid body (six degrees of freedom) affine transformation (Roche, 2011). Following slice timing and motion correction a mean functional image was coregistered to the structural scans using FreeSurfer’s boundary-based coregistration *bbregister* algorithm. Following coregistration, a binarized and dilated by one voxel aparc+aseg mask was used to skull-strip our functional data. Voxel-wise intensity outliers in the fMRI time series were interpolated with the *3dDespike* algorithm from AFNI. Functional data were high pass temporal filtered with a 1/128 Hz threshold. The data were spatially smoothed using the FSL *SUSAN* algorithm with a 5 mm FWHM Gaussian kernel. This preserves an image’s underlying structure (tissue types) by averaging the central voxel with local voxels, which have similar intensities (Smith and Brady, 1997). Thus, localization specificity is optimized and uncorrelated noise is reduced. Functional volumes with global intensities exceeding 3 standard deviations of the mean time series or greater than 1 mm composite (the Euclidian combination of head translations and rotations) framewise displacement were flagged as outliers by ART to be regressed out of the first-level design matrices—a separate column for each outlier consisting of zeros and a one at the flagged time point.

To mitigate group differences related to registration errors and optimize spatial normalization we created a study-specific template using default parameters specified in the *buildtemplateparallel.sh* script as implemented in ANTS (Avants et al., 2008). Normalization to a study-specific template offers superior registration over direct pairwise methods (Klein et al., 2010). T1-weighted structural images from 20 participants (10 from the Study group and 10 from the Test group) were skull stripped by multiplying each participant’s orig.mgz Freesurfer file by their binarized and one voxel dilated FreeSurfer segmentation mask (aparc+aseg.mgz). When building our study template, we implemented a rigid-body registration (six degrees of freedom) with each participant’s T1-weighted scan to the Montreal Neurological Institute (MNI) template using FSL’s *FLIRT* algorithm. This first pass establishes our template generation close to a common reference frame (MNI space) and mitigates large spatial shifts across participants resulting from differences in positioning at the time of data collection. Following template generation, each participant’s original skull stripped brain was normalized to the study-specific template *via* the non-linear symmetric diffeomorphic mapping implemented in ANTS, using the default parameters specified in the *antsIntroduction.sh* script. The resulting transformation matrices from co-registration and normalization were concatenated and applied to contrast parameter estimates before group-level analyses.

#### fMRI Data Analysis

Data analysis was performed in FSL according to a general linear model approach. First-level models included both performance (remembered, forgotten, and incorrect responses) and nuisance (six motion parameters and outlier volumes identified by ART) regressors. Task performance regressors were convolved with FSL’s double gamma hemodynamic response function with a 6 s duration (4 s of the word-pair test presentation, Swahili word, and the first letter of the English translation (e.g., “vuke-s”) plus 2 s of a fixation-cross presentation when the participants were instructed to say the English word (e.g., “steam”) or if they could not remember the translation, “forget”). We elected to convolve over the cue period (4 s) and when responses were made (2 s) for the following reasons: during the training portion of the experiment on day 1 both the Study and Test groups were required to read aloud the Swahili-English word pairs. Thus, we believe that motor responses constitute an important component in the mnemonic representation. Further, and perhaps more importantly, the only time point in which we can be certain cued recall occurred was following the response, thus to capture the variability in the underlying processes we convolved over a wider period despite the potential for additional factors (e.g., motion related to speaking, decision making, and motor preparation), which were matched across both groups, to account for our observed results. Thus, caution is warranted in the interpretation of the group differences given the wide convolution window (6 s) utilized. The contrasts of interest were remembered greater than forgotten and forgotten greater than remembered. Resulting beta images and variance files were concatenated across runs and analyzed with a weighted fixed-effects model using FSL’s *flameo* to obtain a within-participant beta estimation. Group-level analyses were performed using a mixed-effects general linear model approach using FSL’s *flameo*, where participants were the random effect and contrasts were the fixed effect. We corrected for multiple comparisons at the voxel level using FSL’s *cluster* algorithm (corrected α < 0.05) using an uncorrected height threshold of *z* > 2.3. Lastly, we performed a conjunction analysis using the *easythresh_conj* script (Nichols, 2007) to evaluate shared regions in which both groups (Test and Study) exhibited performance-related effects.

## Results

### Behavioral Results

#### Tests During Training Outside of the Scanner

**Figure 2:**
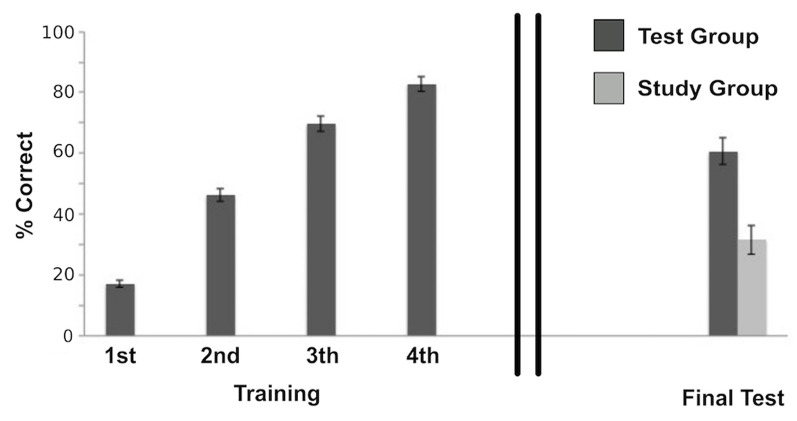
Behavioral performance of the Test group during the initial training session, which shows accuracy improvement across Test sessions (*F*_(3,60)_ = 231.17, *p* < 0.001); and final test performances of both the Study group and the Test group after a 1-week delay, that shows a significant difference between groups (*t*_(39)_ = 4.52, *p* < 0.001; Cohen’s *d* = 1.44) with a retrieval success twice as high for the Test group compared to the Study group. Error bars represent ± standard error of the mean.

We analyzed Test Group performance using a repeated measure ANOVA with correct recall percentage as the dependent measure. Participants’ accuracy improved significantly across Test sessions (*F*_(3,60)_ = 231.17, *p* < 0.001; Figure 2). This was best fit by a linear trend (*F*_(1,20)_ = 300.93, *p* < 0.001; generalized η^2^ = 0.92). A similar performance measure was not available for the Study group given the experiment design.

#### Test in the Scanner

Correct performance in the scanner following a 1-week delay was assessed for each group: in the Study group, a mean of 18.85 of the total 60 word-pairs (*M* = 31.62%, *SD* = 21.42) were correctly recalled, whereas in the Test group 36.38 of the 60 word-pairs (*M* = 60.63%, 232 *SD* = 19.72) were correctly remembered. Thus, retrieval success was about twice as high for the Test group as compared to the Study group. As to be expected, this difference was significant (*t*_(39)_ = 4.52, *p* < 0.001; Cohen’s *d* = 1.44).

### Neuroimaging Results

An overview of the neuroimaging results is given in Table 1.

**Table 1:** Peak intensity and coordinates in Montreal Neurological Institute (MNI) space of within and between-group functional magnetic resonance imaging (fMRI) comparisons.

		MNI space coordinates		
Region	*z*-values	*x*	*y*	*z*
**Between group Remembered > Forgotten**
Test Group > Study Group				
Left putamen	4.16	−29	−1	10
Left supramarginal gyrus	3.55	−59	−33	41
Study Group > Test Group				
Left Insula/DLPFC	4.10	−30	24	−3
Right Insula/DLPFC	4.33	27	23	−3
Left medial frontal gyrus	3.42	−1	32	40
**Within group Remembered > Forgotten**
Study group only
Left paracingulate gyrus (medial prefrontal)	5.49	−6	48	4
Right frontal operculum/inferior frontal gyrus	4.88	47	15	7
Left angular gyrus	4.88	−44	−57	32
Left middle temporal gyrus	4.69	−62	−23	−6
Left precentral gyrus	4.33	−58	2	11
Test group only
Right supramarginal gyrus	5.48	56	−23	40
Left supramarginal gyrus	6.16	−56	−33	38
Left posterior cingulate gyrus/precuneous (parietal)	5.68	−7	−43	38

#### Between Groups (Study vs. Test)

**Figure 3:**
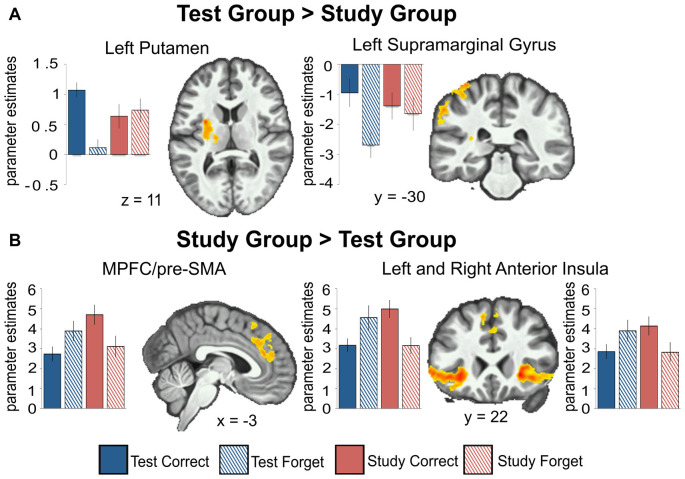
Between-group comparisons show regions with greater activation for the Test group compared to the Study group **(A)** and for the Study group compared to the Test group **(B)** for remembered (Correct) greater than forgotten (Forget) word-pairs. Beta weights are plotted for each group across all conditions (Test group remembered, Test group forgotten, Study group remembered, Study group forgotten) and regions. The left putamen (*t*_(20)_ = 7.49, *p* < 0.001) and left inferior parietal cortex near the supramarginal gyrus (*t*_(20)_ = 5.47, *p* < 0.001) show correct retrieval effects for the Test group; while the opposite is observed in the right insula/dorsolateral prefrontal cortex (DLPFC; *t*_(19)_ = 3.76, *p* = 0.001); MPFC/pre-SMA (*t*_(19)_ = 3.817, *p* = 0.001) and left insula/DLPFC (*t*_(19)_ = 6.30, *p* < 0.001) for the Study group. All activations are shown with an uncorrected height threshold of *z* ≥ 2.3 corrected for multiple comparisons at the cluster level resulting in an overall corrected alpha of *p* < 0.05. Error bars represent ± standard error of the mean.

We examined the interaction between groups (Test vs. Study) and accuracy (remembered vs. forgotten). The left putamen and left inferior parietal cortex near the supramarginal gyrus (we will refer to this region as the supramarginal gyrus for brevity throughout) showed correct retrieval effects in the Test Group compared to the Study Group (Figure 3A).

The dorsal MPFC/pre-SMA, bilateral frontal operculum extending into the dorsolateral prefrontal cortex (DLPFC), and bilateral anterior insula showed an elevated effect of correct retrieval in the Study Group than in the Test Group (Figure 3B). In the Test Group, the opposite pattern was observed—there was greater activation for forgotten than remembered word-pairs in the same regions. Thus, regions throughout the lateral and medial frontal cortex that showed greater activation for remembered vs. forgotten word-pairs in the Study group showed the reverse pattern in the Test group.

To illuminate the specific interaction pattern further, we extracted beta weights for each condition (correct vs. forget) and group (test vs. study; graphically depicted in Figure 3).

#### Within Groups (Study and Test)

We examined performance-related activations in each group separately. In the Study Group, there were significant effects when comparing remembered vs. forgotten trials throughout a wide network of regions—the bilateral medial temporal lobe (including the hippocampus), DLPFC, ventrolateral prefrontal cortex, anterior insula, parietal cortex, lateral temporal cortex, medial prefrontal cortex, and posterior cingulate/precuneus (Figure 3). In the Test Group, there was a similar pattern of correct retrieval effect. However, the Test Group also exhibited successful performance-related effects in bilateral putamen. Additionally, the Test group showed greater activation for forgotten than remembered trials in the dorsolateral and ventrolateral prefrontal cortex, anterior insula, and dorsal medial prefrontal cortex/pre-supplementary motor area (Table 1). No region showed greater activation for forgotten than remembered word-pairs in the Study or Test Groups.

**Figure 4:**
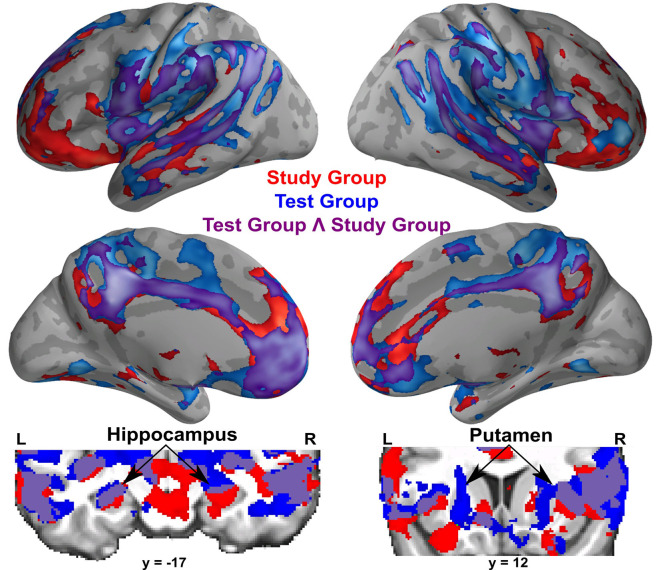
Within-group activations for successful memory retrieval contrasting remembered greater than forgotten word-pairs in the Study group (in red), the Test group (in blue), and the conjunction of the Study and Test groups (in purple). Coronal images show subcortical activations, specifically the hippocampus and the putamen. All activations are shown with an uncorrected height threshold of *z* ≥ 2.3 correcting for multiple comparisons at the cluster level resulting in an overall corrected alpha of *p* < 0.05.

A conjunction analysis revealed that both groups had significant activation overlap for remembered compared to forgotten word-pairs in bilateral superior and middle temporal gyrus (including the hippocampus), parietal cortex including the postcentral gyrus, precuneus/posterior cingulate gyrus, insular cortex, and frontal cortex including the frontal pole, medial prefrontal cortex, and anterior cingulate gyrus (Figure 4).

## Discussion

In the present study, we investigated the neural basis of the testing effect by comparing fMRI activity at the final retrieval test for two groups: one which learned word-pairs *via* repeated study and another which learned *via* repeated retrieval practice. Results showed that there was a distinct functional brain activation pattern during the final test for each group. Behaviorally, after a week, the Test Group exhibited a doubling of long-term memory relative to the Study Group despite an equal number of presentations during training. Brain differences were observed during retrieval based on different learning experiences during encoding: studying and testing (Test Group) vs. studying only (Study Group). Following an interaction analysis, the Test Group exhibited greater activation for successful retrieval in the left putamen and inferior parietal cortex near the supramarginal gyrus, whereas the Study Group exhibited no performance-related activations in the same regions. The Study Group, on the other hand, exhibited an effect of successful retrieval in the dorsal MPFC/pre-SMA, bilateral frontal operculum extending into the DLPFC, and bilateral anterior insula, whereas the Test Group exhibited the opposite pattern of performance-related activation in the same regions. Thus, brain activation differences associated with the testing effect on retrieval were not simply variations of activation magnitudes in a common neural network, but rather involved the contribution across memory systems, reflected in the greater activations in the putamen and supramarginal gyrus in the Test Group, and potentially distinct strategic retrieval mechanisms between groups, evinced by the reversal in performance-related activations in the Test Group relative to the Study Group.

### Unique Activations Associated With Repeated Retrieval

Only the Test Group exhibited greater activations associated with successful retrieval in the left putamen and left supramarginal gyrus. The left-lateralization of these differences may be related to the verbal nature of the learning task. Further, the engagement of the putamen may be related to the sensorimotor nature of the reading-aloud encoding tasks. The putamen activation provides evidence for cooperative contributions between memory systems during associative retrieval. This is consistent with observed activation differences in the basal ganglia using a similar task during encoding (Van den Broek et al., 2013). The putamen is often implicated in procedural learning (Grafton et al., 1992; Miyachi et al., 1997, 2002; Jueptner and Weiller, 1998; Turner et al., 2003). It receives somatotopically-organized projections from the sensorimotor cortex (DeLong and Georgopoulos, 1981; Alexander et al., 1986), and there is a modulation in the firing rate of its neurons during the acquisition, extinction, and re-emergence of procedural learning (Barnes et al., 2005). Previous neuroimaging studies have also observed putamen activation during speech planning and production tasks (Price, 2010). Activations in the putamen marking successful retrieval unique to the Test Group may reflect procedural contributions related to speech planning and production, acquired during the testing at encoding. This may aid associative memory through the integration of sensorimotor related regions into a memory network for successful verbal recall. Similar cooperation between memory systems was previously identified during an episodic encoding task (Sadeh et al., 2011).

The unique activation of the inferior parietal cortex near the supramarginal gyrus during successful verbal recall in the Test Group may reflect this region’s multifaceted contribution to episodic retrieval. The supramarginal gyrus is located in the posterior parietal cortex anterior to the angular gyrus. Posterior parietal cortex activations have consistently been observed during episodic retrieval tasks (Hutchinson et al., 2009; Myskiw and Izquierdo, 2012). Activations in the posterior parietal cortex during retrieval have been correlated with the vividness (Wheeler and Buckner, 2004) and confidence (Kim and Cabeza, 2007) of recalled memories. This region is also thought to play an important role in the attentional capture by relevant memory cues (Ciaramelli et al., 2008). While, in our study, the supramarginal gyrus showed greater activations for remembered vs. forgotten word pairs, the observed differences across conditions were below the baseline. We believe these results are a product of using an active baseline condition during data acquisition (Stark and Squire, 2001) and may reflect the role of the supramarginal gyrus during arithmetic, language, and phonological processing tasks (Hartwigsen et al., 2010; Evans et al., 2016). Posterior parietal cortex activations have consistently been observed during similar studies of the testing effect during encoding (Eriksson et al., 2011; Nelson et al., 2013; Wing et al., 2013; Van den Broek et al., 2013; Keresztes et al., 2014; Liu et al., 2014; Vestergren and Nyberg, 2014; Wirebring et al., 2015) and retrieval (Keresztes et al., 2014; Wirebring et al., 2015). One study noted decreased pattern similarity across test repetitions during encoding in the parietal cortex, which the authors posited contributed to processes such as semantic elaboration (Wirebring et al., 2015). Thus, activations in the inferior parietal cortex near the supramarginal gyrus may reflect enhanced attentional capture by memory cues, increased memory strength or confidence through the creation of more elaborated semantic memories, or augment neural representations for word-pairs through the integration of speech-related semantic regions with prototypical episodic memory-related regions. In summary, we contend that the testing effect partially arises from retrieval practice engaging widespread memory-related neural areas that represent the cooperation between different memory systems. Further research is necessary to disambiguate the relevant contributions to the testing effect of these different yet potentially related processes.

### Activations Associated With Repeated Study

Increased activations for successful cued-recall in the bilateral insula/frontal operculum extending into the DLPFC and bilateral medial frontal gyrus/pre-SMA may reflect greater reliance on top-down executive control of successful retrieval in the Study Group relative to the Test Group. Frontal activations have consistently been observed during successful declarative retrieval (Rugg et al., 1996; Buckner et al., 1998; McDermott et al., 2003; Simons and Spiers, 2003; Spaniol et al., 2009; Huijbers et al., 2013). These regions have been correlated with top-down control operations often employed during retrieval (Simons and Spiers, 2003; Badre and Wagner, 2007) and may be related to item-specific search operations (Makino et al., 2004; Long et al., 2010). Research in non-human primates demonstrated that top-down signals from the frontal cortex are important for the executive control of voluntary recall from long-term memory (Tomita et al., 1999).

A particularly striking contrast between the two groups occurred in the frontal cortex where the Study Group exhibited greater activations for successful retrieval, whereas the Test Group did so for unsuccessful retrieval. Greater prefrontal activations in the Study Group for correct retrieval could reflect more top-down executive control that leads to successful cued recall. In contrast, the Test Group may not require similar search processes for correctly recalled words, but instead, top-down search processes were used for words that were not as accessible and ultimately forgotten. This reduced reliance on the frontal cortex for successful retrieval in the Test Group is consistent with results from Wirebring et al. (2015). This study showed reduced prefrontal cortex activation during repeated correct retrieval at the encoding phase tests, only for words subsequently remembered but not subsequently forgotten at the final test. Thus, successful retrieval at the final test is related to reduced prefrontal activation during repeated correct retrieval during encoding. Our findings regarding the decreased activation of prefrontal areas in the Test Group for correct retrieval at the final test are consistent with the results of Wirebring et al. (2015).

### Activations Associated With Successful Recall in Both Groups

Within-groups results showed similar activations for successful recall in both groups, which recruited medial temporal lobes including the bilateral hippocampus, posterior parietal cortex, precuneus, and prefrontal cortices. The Test Group also showed greater activation for successful recall in bilateral putamen, which is consistent with a study examining successful recall for studied and tested word pairs (Wirebring et al., 2015). A conjunction analysis confirmed the large overlap in activations between groups for remembered vs. forgotten trials, involving regions typically identified during successful retrieval (Buckner et al., 1998; Spaniol et al., 2009). These results suggest that both groups activated prototypical memory-related regions during successful retrieval.

### Nature of Retrieval Practice

The testing effect is a behavioral phenomenon, evident across different sensory modalities, types of memory (e.g., declarative vs. procedural), and learning contexts. Thus, it is likely that the benefits of the testing effect arise from a variety of underlying mechanisms. For example, most testing effect studies have used verbal materials. However, the benefits of testing have been shown to support non-verbal visual and spatial information, including locations on maps (Carpenter and Pashler, 2007; Rohrer et al., 2010), identifying birds (Jacoby et al., 2010), name-face associations (Morris and Fritz, 2002; Helder and Shaughnessy, 2008), statistics (Szpunar et al., 2013), and spatial locations of objects (Sommer et al., 2008). Retrieval benefits have also been found with non-declarative tasks, including resuscitation skills learning (Kromann et al., 2009) and inductive learning of input-output functions (Kang et al., 2011). Future research is needed to identify common mechanisms across the disparate methodological implementations.

A potential criticism of the current study and similar retrieval practice paradigms is that Test Group participants, while never explicitly told, could guess during training the final test format and adapt their learning to that specific test. Thus, the testing effect would not reflect the influence of retrieval practice, but rather transfer-appropriate processing from the training to the final test. There is evidence, however, that such a narrow study-test matching is an unlikely explanation of the testing superiority. Studies examining this question showed that the testing effect was maintained when the test format was changed between training and final test (e.g., from free recall to recognition; Carpenter and DeLosh, 2006), or from short-answer questions to multiple-choice questions (Kang et al., 2007; for a review, Pan and Rickard, 2018).

The memory advantage of the Test Group in the current study could be interpreted as reflecting encoding specificity in which vocalization processes used during encoding were transferred and facilitated the final test performance. However, this is unlikely given that both groups vocalized word-pairs during the training. The Test Group produced fewer correct vocalizations of word-pairs because of errors made during training, while the Study Group successfully vocalized the correct English translation with every presentation.

Another effect related to retrieval practice is the behaviorally well-defined “generation effect” in which active information production improves memory performance (Slamecka and Graf, 1978). While both effects share similar surface-level features, upon closer inspection they are dependent on different processes and likely unique neural substrates. For example, the testing effect is related to new information acquisition, while the generation effect is dependent on the reactivation of well-learned associations and rules (e.g., production of synonyms, categories, rhymes, multiplications; Bertsch et al., 2007).

## Conclusion

As in prior behavioral studies, using testing during encoding, despite equated learning time with repeated study only, yielded a great increase in long-term memory, here doubling recall after a 1-week delay. The present findings suggest that for such verbal learning associated with testing during encoding, a left-hemisphere striatal/parietal cortex facilitated potent correct retrieval at the final test. In contrast, learning based on repeated study only appears to have a much greater dependence on prefrontal regions that may have supported effortful search processes in long-term memory. These findings indicate that a unique neural network was engaged at retrieval that reflected differential kinds of learning at study.

## Data Availability Statement

The raw data supporting the conclusions of this article will be made available by the authors, without undue reservation.

## Ethics Statement

The studies involving human participants were reviewed and approved by Massachusetts Institute of Technology Committee on the Use of Humans as Experimental Subjects. The patients/participants provided their written informed consent to participate in this study.

## Author Contributions

EM-G, AM, and JG have designed and conducted this study. They were involved in data collection, data analysis, and writing of the manuscript. All authors contributed to the article and approved the submitted version.

## Conflict of Interest

The authors declare that the research was conducted in the absence of any commercial or financial relationships that could be construed as a potential conflict of interest.
